# Factors influencing fear of falling in community-dwelling older adults in Singapore: a cross-sectional study

**DOI:** 10.1186/s12877-022-02883-1

**Published:** 2022-03-07

**Authors:** Jacqueline Giovanna De Roza, David Wei Liang Ng, Blessy Koottappal Mathew, Teena Jose, Ling Jia Goh, Chunyan Wang, Cindy Seok Chin Soh, Kar Cheng Goh

**Affiliations:** 1 National Healthcare Group Polyclinics, 3 Fusionopolis Link #05–10, Nexus@one-north, 138543 Singapore, Singapore; 2 National University Health System, 5 Lower Kent Ridge Rd, 119074 Singapore, Singapore

**Keywords:** Older adults, Community, Fear of Falling, Frailty, Falls

## Abstract

**Background:**

Fear of falling (FoF) has far-reaching implications including activity restriction, functional decline and reduced quality of life. It is a common consequence of falls but may be present even in non-fallers. This study aimed to determine the factors associated with FoF in a segment of Singapore’s community-dwelling older adults.

**Methods:**

This descriptive cross-sectional study recruited a convenience sample of adults aged 65 and above from 4 primary care clinics from September 2020 to March 2021. Data were collected on demographic factors, clinical factors such as multi-morbidity, falls characteristics such as history of falls, injuries, and reasons for falls and frailty as determined by the Clinical Frailty Scale (CFS). FoF was measured using the Short Falls Efficacy Scale–International (Short FES-I), cut-off score of 14 and above indicated high FoF. Logistic regression was used to determine factors associated with high FoF.

**Results:**

Out of 360 older adults, 78.1% were Chinese and 59.7% females. The mean age was 78.3 years and 76 (21.1%) had a history of falls in the past six months. Almost half (43.1%) were mildly to moderately frail and most (80.6%) had multi-morbidity. The mean FoF score was 15.5 (SD 5.97) and 60.8% reported high FoF. There were statistically significant differences in age, gender, ethnicity, marital status, educational level, use of walking aid, multi-morbidity, frailty status, history of falls within six months and reason for falls between patients who had high FoF versus those who had moderate or low FoF. Logistic regression found that Malay ethnicity (OR = 5.81, 95% CI 1.77–19.13), marital status, use of walking aids (OR = 3.67, 95% CI = 1.54–8.77) and frailty were significant factors associated with high FoF. Compared to those who were never married, the odds of high FoF were significantly higher in married older adults (OR = 6.75, 95% CI 1.39 to 32.76), those who were separated or divorced (OR 10.40, 95% CI 1.13 to 95.76) and those who were widowed (OR = 7.41, 95% CI 1.51 to 36.41). Compared to well older adults, the odds of high FoF were significantly higher in pre frail older adults (OR = 6.87, 95% CI = 2.66–17.37), mildly frail older adults (OR = 18.58, 95% CI = 4.88–70.34) and moderately frail older adults (OR = 144.78, 95% CI = 13.86–1512.60).

**Conclusions:**

The study found that pre frail to moderately frail older adults as determined by CFS have significantly higher risk of high FoF. The demographic factors such as marital status and ethnicity and falls characteristics associated with FoF in this study will be helpful to develop targeted and tailored interventions for FoF.

## Background

Fear of Falling (FoF) is an internal psychological phenomenon of concern or anxiety about falling [1]. It may be sequelae of falling, and was originally called post-fall syndrome, but may also occur in the absence of fall history. FoF is common amongst older adults and has serious consequences. It leads to downward spiral of activity restriction and functional decline [2–4]. A higher FoF was found to lower quality of life through increased social isolation over time [5]. Various demographic and clinical factors influence FoF. Notably, the risk of FoF is higher amongst patients aged 75 years or older, female, lower educational level, and medical conditions such as diabetes mellitus, arthritis or depression [6]. Internationally, ethnicity has not been well studied as a risk factor for FoF or has not been found to be a significant factor for FoF [7].

FoF is described as both a cause and consequence of falls [8]. Patients who had experienced a previous fall were 2.5 times more likely to report FoF [9]. Similarly, a high score on the Fear of Falling Avoidance Behaviour Questionnaire was predictive of falls at one year [10]. Falls have potentially serious complications and long-term sequelae. Falls is the commonest mechanism of injury in older adult patients presenting to hospitals, and complications such as a hip fracture leads to increased disability in 20 to 60% of patients post fracture [11]. Intrinsic factors like older age, increasing number of chronic diseases, analgesia use, use of mobility aids, eye diseases, and loss of foot sensation and environmental factors such as slippery bathrooms were associated with an increased fall risk [12]. Despite the association of falls with FoF, details about falls such as location and reasons for falls in association with FoF have not been explored. The use of various types of walking aids in association with FoF has also not been well studied.

Frail older adults are at a higher risk of falling [13, 14]. Frailty is described as a clinical syndrome where a loss of physiological reserves leads to an increase in adverse events from relatively minor stresses [15]. The prevalence of frailty increases with age, female gender, low education, low socioeconomic status, multi-morbidity, pain and polypharmacy [16, 17]. Studies have shown frailty is a strong risk factor for FoF. A higher proportion of frail patients have FoF compared to those who were pre-frail [18]. There was a higher proportion of pre-frail and frail patients with FoF compared to those who were not frail [7]. In these studies, the frailty scales used were the frailty phenotype and the FRAIL scale. Many scales have been developed to measure frailty in patient cohorts. The Clinical Frailty Scale (CFS) is a frailty scale that has high feasibility for use for assessment of frailty in primary care given its short time for assessment [19]. The CFS has been shown to correlate well with an increased risk of falls [20]. The CFS has not been widely used in studies of FoF.

While a recent study in Singapore found a significant association between frailty and FoF, the proportion of frail participants was small (3.4%) and pre-frail and frail older adults were thus grouped together for analysis [7]. This limited the analysis of FoF in increasing frailty states such as moderate frailty. Further, the study utilized only a single question for FoF [7]. Thus, this study planned to address the gaps by focusing on FoF and frailty using a validated questionnaire for FoF, namely the Falls Efficacy Scale – International (FES-I); and CFS as a measure for frailty. This study also planned to further explore demographic factors such as ethnicity which had limited previous studies as well as falls characteristics which were not explored in depth previously. The multi-racial population in Singapore created an opportunity for our team to determine the impact of ethnicity on FoF.

## Methods

### Aims

The aim of the study was to determine the factors associated with high FoF in community-dwelling older adults, particularly the association between frailty, falls characteristics, demographic factors and FoF.

### Study design

This was a descriptive cross-sectional study utilizing interviewer-administered questionnaires.

### Setting and participants

Convenience sampling was used. Participants were recruited on a case encounter basis from four public primary care clinics in Singapore from October 2020 to March 2021.

Older adults aged 65 and above, who had chronic disease management follow-up with the primary care clinics, and able to communicate in English or major local languages, Mandarin, Malay and Tamil were eligible. Participants unable to agree to participation or answer the questionnaire due to cognitive impairment as determined by referring clinicians; severely frail or very severely frail with a Clinical Frailty Scale (CFS) score of 7 or more were excluded. Cognitive impairment was defined as a diagnosis of dementia in their medical records. The severely and very severely frail were excluded as their ability to perform activities associated with fear of falling such as stair climbing or walking on slopes was expected to be limited due to complete dependence for personal care.

### Data collection

Potential participants were identified on a case encounter basis by their attending healthcare providers when they attended at the clinics for follow-up visits. Healthcare providers referred all potential participants who met the inclusion criteria to study team members. Questionnaires were administered by study team members proficient in participants’ spoken language after study was explained and verbal consent taken. Training was provided to study team members to ensure consistent administration of the questionnaire including determination of CFS and FES-I. No potential participants declined participation in the study. To reduce bias, study team members did not administer questionnaires for their own patients but referred them to other study team members instead. In view of the ongoing pandemic situation, participants were given the option of having the questionnaire administered via telephone at a mutually convenient time, however, none requested for this option. All questionnaires were complete as they were interviewer-administered by study team and participants answered fully.

The questionnaires consisted of demographic and clinical data on participants’ age, gender, ethnic group, marital status, educational level, co-morbidities, frailty status, falls history and characteristics, and use of walking aid. The list of chronic conditions was derived from a list developed to document self-reported chronic conditions in primary care [21]. Multi-morbidity was defined as the coexistence of three or more chronic conditions in an individual [22].

Frailty status was determined by the Clinical Frailty Scale (CFS), a judgement-based tool to assess the overall level of fitness and function of older adults, with levels ranging from 1 (very fit) to 9 (terminally ill). Score 4 indicates vulnerable, score 5 indicates mildly frail and score 6 indicates moderately frail [23]. CFS was highly correlated (r = 0.80) with the Frailty Index [23]. A recent scoping review revealed that the CFS has been widely used in multiple settings and has good association with clinical outcomes [20].

A fall was defined as ‘a sudden, unintentional change in position causing an individual to land at a lower level (either on an object or on the floor) other than as a consequence of overwhelming external force’ [24]. Falls history and characteristics included number of falls in past six months, location of falls, reason for falls and any injuries sustained.

Fear of falling (FoF) was measured using the Short Falls Efficacy Scale – International (FES-I). The Short FES-I is a 7-item measure of “fear of falling” and has been demonstrated to have good reliability and validity [25]. It was also evaluated in Malaysia and showed good internal consistency, test–retest reliability, construct validity, and responsiveness, with Cronbach alpha of 0.905 and intra-class correlation coefficient 0.997 for the English version [26]. Scores are calculated by summing up responses for each item, with a range from minimum 7 (no concern about falling) to maximum 28 (severe concern about falling). A score of 7 to 8 points indicates low concern, 9 to 13 indicates moderate concern and 14 to 28 indicates high concern. Permission to use Short FES-I was obtained from Professor Chris Todd https://sites.manchester.ac.uk/fes-i/.

The questionnaire was reviewed by an expert panel comprising multidisciplinary members of the Geriatrics Specialty Advisory Group including a physiotherapist, and external geriatric trained doctors and nurses. The questionnaire was piloted on community-dwelling older adults with chronic conditions attending the four participating clinics and at various levels of frailty prior to implementation.

### Data analysis

SPSS version 27 was used to analyze the data. Power analysis for logistic regression, calculated based on the work of Peduzzi et al. [27] for 17 covariates, assuming 0.5% of positive cases in the population and 10% drop-out rate, at least 358 participants would be required.

Demographic and clinical characteristics, prevalence of falls and prevalence of FoF were reported using descriptive statistics. Chi-square was used to compare differences in FoF between categorical variables. These variables were age, gender, ethnicity, marital status, highest education, whether they lived alone, presence of multi-morbidity, frailty category, use of walking aid, falls in past six months, injurious falls, location of falls and reasons for falls. However, if there was more than 20% of expected frequency less than 5 and for 2 × 2 table, Fisher exact test was used. FoF comparisons were performed prior to logistic regression and significant variables were included in the logistic regression model. Some variables such as use of walking aid were collapsed into fewer categories for logistic regression due to small sample sizes for some response options. Variables with small sample sizes such as reason for fall were excluded. Statistical significance was set at a *P*-value less than 0.05.

### Ethical considerations

Ethical approval was obtained from National Healthcare Group Domain Specific Review Board (DSRB Ref: 2020/00847). All methods were performed in accordance with the relevant guidelines and regulations. Potential participants were referred to study team members by attending healthcare professionals after consultation. Trained study team members proficient in participants’ language explained the study and obtained verbal informed consent. Hardcopy data was stored in designated locked cabinets. Electronic data was stored in a secured electronic database. All hard copy or electronic data was accessible to authorized study team members only.

## Results

### Demographic characteristics and high Fear of Falling

The study recruited 360 older adults. The majority (78.1%) were Chinese and 59.7% were females. The mean age was 78.3 years and 76 (21.1%) had a history of falls in the past six months. There were 29.4% categorized as CFS 1 to 3 (well), 27.5% CFS 4 (pre-frail), 23.9% CFS 5 (mildly frail) and 19.2% CFS 6 (moderately frail). Most (80.6%) had multi-morbidity (three or more chronic conditions). 219 respondents (60.8%) had high FoF as defined by short FES-I score between 14 and 28. We found statistically significant differences in age, gender, ethnicity, marital status, educational level, use of walking aid, multi-morbidity, frailty status, history of falls within six months and reason for falls between patients who had high FoF versus those who had moderate or low FoF. The demographic, clinical and falls characteristics and comparison between high and moderate or low FoF are shown in Table 1.

**Table 1:** Participants’ characteristics and Fear of Falling (FoF) (*n* = 360)

Variable	Total n (%)	High FoF, n (%)	Low/ moderate FoF, n (%)	*P*-value
**Age Category**				< 0.001
Young-old (65–74)	131 (36.4)	59 (45.0)	72 (55.0)	
Old-old (75 & above)	229 (63.6)	160 (69.9)	69 (30.1)	
**Gender**				< 0.001
Male	145 (40.3)	72 (49.7)	73 (50.3)	
Female	215 (59.7)	147 (68.4)	68 (31.6)	
**Ethnicity**				0.031
Chinese	281 (78.1)	165 (58.7)	116 (41.3)	
Malay	45 (12.5)	36 (80.0)	9 (20.0)	
Indian	31 (8.6)	17 (54.8)	14 (45.2)	
Others	3 (0.8)	1 (33.3)	2 (66.7)	
**Marital Status**				< 0.001
Married	187 (51.9)	93 (49.7)	94 (50.3)	
Widowed	145 (40.3)	109 (75.2)	36 (24.8)	
Separated/ Divorced	15 (4.2)	12 (80.0)	3 (20.0)	
Never Married	13 (3.6)	5 (38.5)	8 (61.5)	
**Highest Education**				< 0.001
No formal education	162 (45.0)	121 (74.7)	41 (25.3)	
Primary	106 (29.4)	60 (56.6)	46 (43.4)	
Secondary	75 (20.8)	31 (41.3)	44 (58.7)	
Tertiary	17 (4.7)	7 (41.2)	10 (58.8)	
**Living Alone**				0.305
No	324 (90.0)	199 (61.4)	125 (38.6)	
Yes	36 (10.0)	20 (55.6)	16 (44.4)	
**Multi-morbidity**				< 0.001
1 or 2 conditions	70 (19.4)	27 (38.6)	43 (61.4)	
3 or more conditions	290 (80.6)	192 (66.2)	98 (33.8)	
**Frailty Category**				< 0.001
Well (CFS 1 to 3)	106 (29.4)	13 (12.3)	93 (87.7)	
Pre frail (CFS 4)	99 (27.5)	61 (61.6)	38 (38.4)	
Mildly frail (CFS 5)	86 (23.9)	77 (89.5)	9 (10.5)	
Moderately frail (CFS 6)	69 (19.2)	68 (98.6)	1 (1.4)	
**Walking aid**				< 0.001
None	155 (43.1)	38 (24.5)	117 (75.5)	
Umbrella	22 (6.1)	15 (68.2)	7 (31.8)	
Walking stick	101 (28.1)	87 (86.1)	14 (13.9)	
Quad stick	25 (6.9)	23 (92.0)	2 (8.0)	
Walking frame	33 (9.2)	33 (100)	0 (0)	
Others	24 (6.7)	23 (95.8)	1 (4.2)	
**Falls in past 6 months**				< 0.001
None	284 (78.9)	155 (54.6)	129 (45.4)	
1 fall	69 (19.2)	59 (85.5)	10 (14.5)	
2 or more falls	7 (1.9)	5 (71.4)	2 (28.6)	
**Fall with injury**^a^				0.059
No	24 (31.6)	23 (95.8)	1 (4.2)	
Yes	52 (68.4)	41 (78.8)	11 (21.2)	
**Location of fall (most recent fall if multiple falls) **^a^				0.056
Outdoors	30 (39.5)	21 (70.0)	9 (30.0)	
Bathroom	18 (23.7)	17 (94.4)	1 (5.6)	
Bedroom	16 (21.1)	16 (100)	0 (0)	
Living room	11 (14.5)	9 (81.8)	2 (18.2)	
Kitchen	1 (1.3)	1 (100)	0 (0)	
**Reason for fall (most recent fall if multiple falls) **^a^				0.013
Trip or slip	29 (38.2)	20 (69.0)	9 (31)	
Lower limb weakness	23 (30.3)	23 (100)	0 (0)	
Giddiness	10 (13.2)	10 (100)	0 (0)	
Lost balance	6 (7.9)	4 (66.7)	2 (33.3)	
Others	8 (10.5)	7 (87.5)	1 (12.5)	

^a^ Excluded those with no falls in past 6 months

There were differences in FoF for age category (Fisher exact, *p* < 0.001). For those 75 years old and above, 69.9% had high FoF and 30.1% had low or moderate FoF; while only 45% of those below 75 had high FoF. For females, 68.4% had high FoF while 49.7% of males had high FoF (Fisher exact, *p* < 0.001). Of those with multi-morbidity, 66.2% had high FoF compared to 38.6% of those who did not have multi-morbidity (Fisher exact, *p* < 0.001).

High FoF was found in older adults of Malay ethnicity (x2 = 8.89, df = 3, *p* = 0.031), separated or divorced (x2 = 27.23, df = 3, *p* < 0.001), had no formal education (x2 = 28.58, df = 3, *p* < 0.001), and were mildly to moderately frail (x2 = 175.94, df = 3, *p* < 0.001).

There were differences in FoF for history of falls in the past six months (x2 = 22.625, df = 2, *p* < 0.001). For those who had fallen once in the past six months, 85.5% had high FoF while 14.5% had low or moderate FoF. For those who had two or more falls, 71.4% had high FoF while 28.6% had low or moderate FoF. For those who had no falls in the past six months, 54.6% had high FoF while 45.4% had low to moderate FoF.

There was no significant difference in FoF for falls with injuries versus those without injuries or in location of fall. The most common reason for falls was trip or slip (38.2%). There were significant differences in FoF for various reasons for falls (x2 = 12.710, df = 4, *p* = 0.013). 69% of those who stated they tripped or slipped had high FoF while 100% of those fell due to lower limb weakness or giddiness had high FoF.

The types of walking aids used included umbrella, walking stick, quad stick, walking frame and others such as shopping trolley as aid. There were significant differences in FoF for types of walking aid (x2 = 157.223, df = 5, *p* < 0.001). While only 24.5% of those who did not use any walking aids had high FoF, 68.2% of those who used umbrellas as aids, 86.1% of those who used walking sticks, 92% of those who used quad sticks and 100% of those who used walking frames had high FoF.

### Factors associated with high Fear of Falling

A multi-variate analysis was performed to identify factors associated with high FoF and to adjust for potential confounders. The logistic regression model to ascertain the effects of age, ethnicity, gender, marital status, educational level, multi-morbidity, CFS category, use of walking aids and falls within six months on the likelihood that participants had high FoF was statistically significant, χ2 [17] = 236.64, *p* < 0.001. Nagelkerke R2 indicated that this model explained 65.3% of the variance and correctly classified 85.6% of cases.

Compared to Chinese, Malay older adults had significantly higher odds of high FoF (OR = 5.81, 95% CI 1.77–19.13). Compared to those who were never married, the odds of high FoF were significantly higher in married older adults (OR = 6.75, 95% CI 1.39 to 32.76), those who were separated or divorced (OR 10.40, 95% CI 1.13 to 95.76) and those who were widowed (OR = 7.41, 95% CI 1.51 to 36.41).

Use of walking aid and frailty were significant factors associated with high FoF. The odds of high FoF were significantly higher in older adults who used walking aids (OR = 3.67, 95% CI = 1.54–8.77). Compared to well older adults, the odds of high FoF were significantly higher in pre frail older adults (OR = 6.87, 95% CI = 2.66–17.37), mildly frail older adults (OR = 18.58, 95% CI = 4.88–70.34) and moderately frail older adults (OR = 144.78, 95% CI = 13.86–1512.60). The logistic regression model is described in Table 2.

**Table 2:** Factors associated with high Fear of Falling from logistic regression model

Explanatory variables	High Fear of Falling		
	**Exp(B)**	**Sig**	**95% CI for EXP (B)**
**Ethnicity**			
Chinese	**Reference**		
Malay	5.817	0.004	1.769–19.125
Indian	0.871	0.813	0.277–2.735
Others	0.142	0.380	0.002–11.177
**Age Category**			
Old-old (75 years & above)	**Reference**		
Young-old (65 to 74 years)	0.860	0.682	0.418–1.770
**Gender**			
Female	**Reference**		
Male	1.117	0.768	0.535–2.334
**Marital Status**			
Never married	**Reference**		
Married	6.750	0.018	1.391–32.756
Separated/ divorced	10.400	0.039	1.130–95.760
Widowed	7.407	0.014	1.507–36.410
**Educational Status**			
Tertiary education	**Reference**		
No formal education	0.859	0.847	0.184–4.007
Primary education	0.446	0.290	0.100–1.992
Secondary education	0.606	0.522	0.131–2.806
**Multi-morbidity**			
1 or 2 chronic conditions	**Reference**		
3 or more chronic conditions	1.149	0.754	0.483–2.730
**Frailty Category (CFS)**			
Well (CFS 1 to 3)	**Reference**		
Pre frail (CFS 4)	6.873	0.000	2.663–17.736
Mildly frail (CFS 5)	18.583	0.000	4.882–70.737
Moderately frail (CFS 6)	144.777	0.000	13.857–1512.600
**Use of Walking Aid**			
No	**Reference**		
Yes	3.673	0.003	1.539–8.765
**Falls within 6 months**			
No	**Reference**		
Yes	2.417	0.056	0.979–5.966

## Demographic characteristics and frailty status

We observed that frailty status was a very significant factor associated with high FoF and proceeded to investigate whether there were differences between groups for demographic characteristics and frailty status. We found significant differences in age, gender, marital status, educational level, multi-morbidity, use of walking aid, fall within six months and frailty status.

More mildly to moderately frail older adults were 75 years and above (x2 = 39.82, df = 3, *p* < 0.001), female (x2 = 24.82, df = 3, *p* < 0.001), widowed (x2 = 45.46, df = 9, *p* < 0.001), had no formal education (x2 = 49.96, df = 9, *p* < 0.001), had multi-morbidity (x2 = 35.94, df = 3, *p* < 0.001), had falls within six months (x2 = 28.05, df = 3, *p* < 0.001) and used walking aids (x2 = 243.09, df = 3, *p* < 0.001).

## Discussion

### Prevalence of Fear of Falling

This study found a high prevalence of FoF amongst our respondents. 60.8% of respondents reported high FoF and 21.4% reported moderate FoF. Studies have used various scales to study the prevalence of FoF and reported a wide variation in prevalence of FoF among community-dwelling older adults in different countries, ranging from 19% in the United Kingdom [28] to 81% in China [29]. In Singapore, a 69.2% prevalence of FoF as assessed by a single question was reported [7]. This makes direct comparison across studies and countries challenging.

There are published articles that have used the Falls Efficacy Scale- International (FES-I) to study the prevalence of FoF. A cross-sectional observational study in Japan using the FES-I categorized FoF into three levels (low, moderate, and high) found that 27.3% were categorized as low concern, 47.9% as moderate concern and 24.8% as high FoF [30]. A similar prevalence of FoF compared to our study was carried out in China. Qin et al. found a 81% prevalence of FoF in 5 communities in China [29].

There were differences in demographics across studies which also makes direct comparison of FoF prevalence challenging. Our study population was of older age and comprised of more individuals who were frail and had multimorbidity, which may have accounted for the differences in reported prevalence.

## Relationship between Demographic Factors, Multi-morbidity, Frailty and Fear of Falling

Our study identified that demographic factors including female gender, age more than 75 years, separated, or widowed marital status, and lack of formal education had higher proportions of respondents with high FoF on bivariate analysis.

These identified risk factors are also associated with a higher risk of frailty. He at al showed that increased age, female gender, presence of three or more chronic diseases and disability in activities in daily living were associated with an increased risk of frailty [31]. Unmarried individuals had a 1.88-fold higher risk of being frail compared to married individuals [32]. Similarly, Wang et al. showed that married individuals were less likely to be frail compared to unmarried individuals [33]. A high level of social engagement amongst unmarried individuals was associated with a lower frailty risk. Kang et al. showed that having a frail spouse was associated with frailty [34]. Taken together, one could hypothesise that a widowed individual would have been living with a frail spouse before his or her spouse’s demise. Reduced social interaction after the spouse’s death, leads to a higher prevalence of frailty amongst widowed individuals.

Our study showed that lower education was associated with higher frailty levels and significantly higher FoF scores. Li et al. showed that persons with high school education or above had a 5.6% lower risk of frailty [35]. The evidence for lower education levels and FoF is less clear. A lower educational level was associated with higher FoF [6, 28, 36, 37]. On the other hand, a study in Singapore found no significant association between FoF and years of education [7]. Interventions for FoF would need to be tailored to persons of varying educational levels, such as utilizing pictorial aids.

This study found that those with multi-morbidity had significantly higher FoF than those without multi-morbidity. Multi-morbidity was also significantly associated with frailty. There is a significant overlap between multi-morbidity and frailty, and between multi-morbidity and FoF. Yarnall et al. described similarities in the pathophysiology between multi-morbidity and frailty, where a cumulative accumulation of deficits from multiple chronic diseases leads to failure of multiple physiological and behavioural factors, leading to increased risk of adverse outcomes from relatively minor stressors [38]. Our findings are similar to several other studies which demonstrated that multi-morbidity was associated with an increased risk of FoF [7, 36]. There are specific medical conditions such as hypertension, stroke, diabetes, arthritis or depression which were associated with an increased risk of FoF [6, 37].

Given that most of the above factors were significantly associated with frailty but not associated with FoF on multivariate analysis, our study suggests that the abovementioned factors are associated with frailty, and in turn, frailty is associated with FoF amongst our respondents in our study.

### Factors associated with Fear of Falling

This study found a significant difference in FoF between ethnic groups in Singapore, with Malay ethnicity having significantly higher FoF than Chinese. Malay ethnicity was not found to be significantly different from other ethnicities in frailty status, thus other cultural reasons may account for this difference; which warrants further exploration to tailor interventions culturally. The effect of ethnicity on FoF is not well established. Merchant et al. found no significant differences in FoF between ethnic groups [7]. However, other studies in countries such as the United Kingdom have found that minority ethnic groups have higher risk for FoF [28].

There were differences in odds of high FoF for marital status in this study. Of interest, those who were married had higher odds of high FoF compared to those who were never married, although those who were widowed, divorced or separated had even higher odds. Marital status was not a factor explored in the study by Merchant et al., but social isolation was a significant factor associated with FoF [7]. It is possible that those who were widowed or divorced experienced greater social isolation. Those who were never married could have developed greater independence from a young age and thus had lower FoF. The association between marital status, social isolation and FoF may need to be explored further in future studies.

This study found that frailty was highly associated with FoF. This was similar to other studies. A study of 183 older adults in Spain who had history of falls in past one year found that 88.8% of frail older adults had FoF compared to 62.4% of those who were not frail. Frail participants had 3.18-fold higher risk of FoF than those not frail [39]. Another study of 165 community-dwelling older adults in China found that 60% of participants were frail and 81% reported FoF. Participants with FoF were 7.2 times more likely to be frail [29]. The study in Singapore, which included 493 community dwelling adults above 60 years old, of which 47.9% were pre-frail and only 3.4% were frail, found that those who were pre-frail or frail were 2.17 times more likely to have FoF [7].

Those who used a walking aid had significantly higher odds of high FoF in this study. Similarly, use of walking aid was associated with FoF in a systematic review and a large scale study in UK [28, 40]. Denkinger at al also showed that the use of a walking aid was significantly associated with fall related activity restriction and falls related self-efficacy. A recent study by Birhanie et al. found that those who used of walking aids were nearly 14 times more likely to have FoF [41]. The types of walking aids used were not specified. For our study, it was found that a higher percentage of those who used quad sticks had high FoF as compared to those who used umbrellas or walking sticks and that all of those who used walking frames had high FoF. It is likely that those using walking frames were also more frail. However, it is also unusual that the use of walking frames, which would be expected to provide greater stability, resulted in high FoF. The perspectives of those using walking aids could be further explored in future studies to further understand the significance of walking aid use and its relation to FoF.

This study found differences in FoF for history of falls in bivariate comparisons, although it was not a significant factor in logistic regression. History of falls is a well-known risk factor as persons who have fallen before are likely to develop fear. History of falls was associated with FoF in a systematic review [40], and recent studies in Singapore [7], Vietnam [42], Brazil [43], Korea [36], Thailand [37], Canada [44] and Ethiopia [41]. None of these studies explored reasons for falls. The most common reasons for falls in our study were trip or slip and lower limb weakness. All of those with lower limb weakness or giddiness reported high FoF. The differences were significant in bivariate analysis, however, reason for fall was not included as a variable in multi-variate analysis as the sample size of 76 participants with fall history was too small. The study in Canada explored seeking medical attention for falls as a factor for FoF, but it was not found to be significant [44]. The study in Ethiopia explored injury with fall but it was not a significant factor associated with FoF in bivariate or multivariate analysis [41]. Future studies may consider exploring falls characteristics and FoF in a larger sample, particularly the association between causes of falls and FoF.

FoF and falls also have bidirectional relationship, where FoF predicts future falls. A study of community dwelling older adults in Serbia found that the average Falls Efficacy Scale score was significantly higher in fallers and FoF was an independent risk factor for falling [45]. A longitudinal, prospective study of community-dwelling adults aged above 75 years old in Spain found that 41.7% of those who had reported FoF at baseline had suffered at least one fall 24 months later and that FoF was a risk factor for falls [9]. Future studies should consider longitudinal studies in the Asian context.

However, more than half (54.6%) of those with no history of falls reported high FoF. Similarly, three-quarters of those with FoF had never experienced a fall in the another study [7]. This raises the possibility of a psychological component to FoF. Older adults may also have other reasons for FoF such as history of near falls increasing anxiety levels, frailty or medical conditions reducing their perceived self-efficacy or self-rated health. This may warrant further investigation, and would also suggests a need for psychological-based interventions for FoF.

### Strengths

This study utilized a validated tool to assess FoF scores, thus allowing comparison of scores between groups and across studies where the FES-I was used. This study also recruited a multi-ethnic Asian population which provided insights on FoF scores among different ethnic groups and identified a potential association between Malay ethnicity and FoF which would warrant further exploration. The recruitment of older adults with varying levels of frailty as determined by CFS also enabled representation of FoF among different frailty levels. By analyzing various risk factors for FoF with frailty we have established a complex relationship between various risk factors and frailty and demonstrated the importance of frailty as a risk factor for FoF. We have identified specific causes of falls which may lead to high FoF, warranting further studies.

### Limitations

This study utilized a cross-sectional design, thus temporality of associations such as those between FoF and future falls could not be determined. Longitudinal studies would be better able to determine this. Another limitation of this study is that information was self-reported and based on recall. The use of judgment-based tool CFS may have resulted in some variability in assessing frailty levels, however, this was minimized by training the study team prior to use. Cognitive screening was not performed prior to recruitment, however, patients with known dementia were excluded. The study population had a higher proportion of Chinese compared to Singapore’s local population. The proportion of persons in various stages of frailty was not proportional to our local population and patients who were severely and very severely frail were excluded. Therefore, caution is advised in generalizing the data to the wider population. Lastly, psychological factors such as self-rated health, anxiety and depression levels were not elicited from participants, which might have provided a more in-depth knowledge on the psychological factors affecting fear of falling in our respondents.

## Conclusions

FoF is a prevalent issue in older adults with frailty. The high prevalence indicates an urgent need for interventions to address FoF in older adults to minimize a vicious cycle of activity limitation, social isolation, functional decline and future falls. This study added to the body of evidence of the demographic and clinical factors that influence FoF and addressed the association between various risk factors, frailty and FoF. We have found a strong association between FoF and frailty. The study also found novel findings such as differences in FoF between ethnic groups and reasons for falls. Our study’s findings suggest that interventions for FoF should be tailored to one’s educational level and be culturally relevant to the person with high FoF. Reasons for falls may also be explored further to address the impact specific reasons have on FoF. The relatively high FoF scores in non-fallers and pre-frail older adults suggests that interventions may also be required and customised to improve their self-efficacy levels and psychological well-being.

## Data Availability

The datasets used and/or analysed during the current study are available from the corresponding author on reasonable request.

## Abbreviations

CFS: Clinical Frailty Scale
FES-I: Falls Efficacy Scale – International
FoF: Fear of falling
